# Supplementary material for: Spatio-temporal evolution and distribution characteristics of the high-quality development of China’s insurance industry
Source: PLoS One. 2025 Mar 4;20(3):e0311568. doi: 10.1371/journal.pone.0311568 (PMC11878946; doi:10.1371/journal.pone.0311568)
Supplement: Attachment — Submitted filename: Response_to_Reviewers_auresp_2.docx [file pone.0311568.s005.docx]

Dear Editor,

We sincerely thank you and all reviewers for the critical/valuable feedback. We feel lucky that our manuscript went to these reviewers as the valuable comments from them not only helped us with the improvement of our manuscript but suggested some neat ideas for future studies. Please do forward our heartfelt thanks to these experts.

Based on the comments we received, careful modifications have been made to the original manuscript. All changes were marked in yellow text and “Track Changes”. We hope the new manuscript will meet your standard. Below you will find out point-by-point responses to the comments/questions.

We look forward to hearing from you regarding our submission. We would be glad to respond to any further questions and comments that you may have.

Heng Zhu

(On behalf of all authors)

**Response to comments on “Spatio-temporal Evolution and Distribution Characteristics of the High-Quality Development of China's Insurance Industry (PONE-D-24-01860)”**

**Response to Reviewer 1 Comments**

For this article, I suggest that the author correct it as follows

(a) The derivation of equations (1) to (9) does not seem to be connected to the content
of Table 3 in the article.

Thank you for the reviewer’s suggestions. Formulas (1) through (9) outline the calculation process for the comprehensive index. The results of formulas (1) through (5), which represent the weight coefficients of each sub-indicator, are presented in Table 1. The outcomes of formulas (6) through (9) are included in the High-Quality.dta file. The results displayed in Table 3 were derived using the standard deviation ellipse calculation program available in ArcGIS software, by inputting the results from formulas (1) through (9) into the program. The calculation formula for the basic parameters of the ellipse, as presented in Table 3, is as follows. We have also added these formulas to the article. (see lines 583 to 587 of the revised manuscript):

$$\overline{X}=\frac{\sum_{i=1}^{n} w_{i}x_{i}}{\sum_{i=1}^{n} w_{i}}$$

$$\overline{Y}=\frac{\sum_{i=1}^{n} w_{i}y_{i}}{\sum_{i=1}^{n} w_{i}}$$

$$\tan\theta=\frac{\left( \sum_{i=1}^{n} w_{i}^{2}\tilde{x}_{i}^{2}-\sum_{i=1}^{n} w_{i}^{2}\tilde{y}_{i}^{2} \right)+\sqrt{{(\sum_{i=1}^{n} w_{i}^{2}\tilde{x}_{i}^{2}-\sum_{i=1}^{n} w_{i}^{2}\tilde{y}_{i}^{2})}^{2}+4\sum_{i=1}^{n} w_{i}^{2}{\tilde{x_{i}}}^{2}{\tilde{y_{i}}}^{2}}}{2\sum_{i=1}^{n} w_{i}^{2}\tilde{x_{i}}\tilde{y_{i}}}$$

$$\sigma_{x}=\sqrt{\frac{\sum_{i=1}^{n} {(w_{i}\tilde{x_{i}}\cos\theta-w_{i}\tilde{y_{i}}\sin\theta)}^{2}}{\sum_{i=1}^{n} w_{i}^{2}}}$$

$$\sigma_{y}=\sqrt{\frac{\sum_{i=1}^{n} {(w_{i}\tilde{x_{i}}\sin\theta-w_{i}\tilde{y_{i}}\cos\theta)}^{2}}{\sum_{i=1}^{n} w_{i}^{2}}}$$

$x_{i}$ and $y_{i}$ are the abscissa and ordinate of the area respectively,$\overline{X}$ and $\overline{Y}$ are respectively the abscissa and ordinate of the ellipse center.$\theta$ represents the azimuth angle of the ellipse, that is, the angle from which the long axis deviates from the north axis. $\sigma_{x}$ and $\sigma_{y}$ represents the length along the x and y axes respectively.

(b) In addition, the empirical results in Table 3 do not include the variable self-
attribution test, the single root test, the co-integration test, and other pre-statistical tests, causality tests, etc.

Thank you for the reviewer's suggestions. The standard deviation ellipse method presented in Table 3 is not a regression technique within the field of econometrics. Instead, it is used to analyze the spatial distribution of high-quality development in the insurance industry across the country. As such, there is no relevant test for this method as you mentioned.

(c)In addition, please confirm whether the 2011 and 2012 years of Inclusiveness in
Table 3 should be 2015 and 2020

Thank you for the reviewer’s correction. We sincerely apologize for this oversight, and we will make the necessary revisions to the relevant sections. We appreciate the reviewer's thorough and conscientious review.

(d) There are too few references and it is not detailed whether the formula was created by the author himself or a reference, and the author is asked to explain why formulas (1) to (9) are used, and whether the model has any shortcomings, rather than other more suitable models to analyze

Thank you for the reviewers' suggestions. Currently, empowerment methods are categorized into subjective and objective approaches. Subjective methods include techniques such as expert scoring and the analytic hierarchy process, while objective methods encompass the vertical and horizontal grading method, the CEITIC method, and the entropy weight method. It is crucial to balance the objectivity and the inherent information content of the data. In our research on the spatial distribution of the high-quality development index, we have employed the entropy weight TOPSIS method, as supported by previous studies (Yang[1], Nie[2], Liu[3], etc.). We have also included the relevant references in our article.

we have also expanded the explanations of each model in the text and included the formulas used for calculating the results.

The kernel density estimation method: Lines 475 to 491.

The Standard Deviation Ellipse (SDE) method：Lines 580 to 591.

The traditional Markov chain：Lines 617 to 626.

The spatial Markov chain：Lines 631 to 643.

Moran's Index：Lines 733 to 742.

**Response to Reviewer 2 Comments**

(1) Although the dimensions of "Inclusiveness," "Sustainability," and "Internal-External Structural Coordination" proposed in the paper are valuable, further clarification is needed on how these dimensions are integrated with specific practices in the insurance industry and their manifestations in different insurance products. It is recommended that you provide concrete examples or empirical data to support the practical application of these dimensions.

Thank you for the reviewer's suggestions. In response, we have expanded the section titled "Theoretical Connotations of High-Quality Development of the Insurance Industry" (see lines 206 to 261 of the revised manuscript) as per your recommendation. To better illustrate how these concepts align with the current realities of China's insurance industry, we have included relevant use cases and data.

(2) Enhancement of the Indicator System:

Thank you for the reviewer's suggestions. It is recommended to include an indicator of insurance consumer satisfaction in the index system to more comprehensively assess the high-quality development of the insurance industry. Additionally, each indicator’s selection should be supported by a more robust theoretical basis and empirical evidence. Please add relevant literature support and empirical data to justify the choice of each indicator.

Thank you for the reviewer's suggestions. We considered including customer satisfaction in our indicator system. However, due to the practical limitations, we have focused on constructing a macro-level indicator system for the high-quality development of the insurance industry. Customer satisfaction, being a micro-level indicator, is not compatible with this approach and is also unavailable at the municipal level. In the future, we plan to develop a micro-level indicator system for the high-quality development of the insurance industry, where we will incorporate customer satisfaction as an indicator based on your feedback. In addition, due to the duplication of references for many indicators, and considering the length, only the dimensional indicators are listed as references, while the calculation of the tertiary indicators is reflected in Table 1. We hope the reviewers can understand. The specific content of the selection basis for indicators in the article is as follows (see lines 293 to 314 of the revised manuscript):

(3)Transparency of Data Sources:

Thank you for the reviewer's suggestions. The details of the data sources in the article are as follows（see lines 354 to 378 of the revised manuscript）:

The main data sources include the "Statistical Yearbooks" of various provinces in China from 2010 to 2020, the "Insurance Statistical Yearbook," and the websites of the financial regulatory bureaus of each province. Some data require processing, and the details are as follows: (1) Due to differences in the statistical scope of insurance institutions among provinces in terms of time and space, rendering them incomparable, the number of insurance companies at the provincial level and above is selected as the indicator. The data on the number of insurance companies in each province are sourced from the provincial "Financial Operation Reports" and the regional version of the "Insurance Statistical Yearbook."(2) Data on the number of insurance industry employees are sourced from the Chinese Labor Economic Database in the EPS database, specifically the number of urban insurance industry employees at the end of the year. (3) For some provinces and years with missing insurance amount data, linear interpolation after logarithmic transformation of insurance amounts is conducted to complete the data. (4) Due to the lack of data on the number of legal entities in each province before 2013, the number of industrial enterprises in each province is selected as a proxy indicator based on data availability and comparability. The data are sourced from the Wind database. (5) Data on the number of enterprises held by insurance institutions in each province are sourced from the China National Research Data Sharing Platform (CNRDS), specifically from databases on shareholding of listed companies and basic information of listed companies. (6) Long-term trends are calculated using the HP filter method. Since the selected premium income, claims, and benefits paid are annual data, a parameter λ=100 is chosen based on previous scholars' experiences to calculate the long-term trend. The deviation between the data for the current year and the long-term trend is considered as the deviation data. (7) The number of insurance technology companies is obtained by searching relevant keywords such as "insurance technology" and "internet insurance" (8) The HHI (Herfindahl-Hirschman Index) is calculated based on the market share of premium income of each insurance company in each province, as provided in the "Insurance Statistical Yearbook."

(4) The paper mentions the removal of map images due to copyright issues. It is suggested that you provide more information on data sources, including the timing of data acquisition, processing methods, and a declaration of dataset completeness to enhance the transparency of the research.

Thank you for the reviewer's suggestion. We did not display the map due to concerns regarding map copyright. The shp file of the China map used in our study was obtained from the National Geographic Center of China (<https://www.ngcc.cn/>). After importing the shapefile into ArcGIS software, we generated the basic framework of China's provincial map and then input the relevant data for calculation. Details about the data source, its integrity, and our methodology were explained earlier in the manuscript. The data used to create the chart can be found in the High-Quality.dta file we provided.

(5) Explanation of Analytical Methods:

While the paper employs various analytical methods, the rationale for selecting each method and the conditions for their applicability are not sufficiently explained. Particularly for the Spatial Markov Chain model, it is advised to provide a detailed description of the selection process for model parameters and the interpretation of model results.

Thank you to the reviewers for your comments. In response, we have expanded the explanations of each model in the text and included the formulas used for calculating the results.

The kernel density estimation method:Lines 475 to 491.

The Standard Deviation Ellipse (SDE) method：Lines 580 to 591.

The traditional Markov chain：Lines 617 to 626.

The spatial Markov chain：Lines 631 to 643.

Moran's Index：Lines 733 to 742.

(6)Analysis of Regional Differences:

The results section shows the development levels of the insurance industry in different regions, but the analysis of the causes of these differences is insufficient. It is advised to explore the reasons for inter-regional differences more deeply by considering factors such as regional economy, policy environment, and market demand.

Thank you for the reviewers' comments. In this revision, we have added an analysis of potential reasons for each result, taking into account factors such as economic development level, economic structure, and regional policies.(see lines 512 to 516; Lines 539 to 543; Lines 663 to 667; Lines 750 to 757)

(7) Improvement of the Literature Review:

The literature review section should more precisely identify the shortcomings of existing research and the innovations of this study, avoiding vague descriptions to enhance the clarity and relevance of the literature review.

Thank you to the reviewers for their comments. In this revision, we have expanded the "Introduction" section (see lines 61 to 95 of the revised manuscript) to include a discussion of the existing literature on measuring high-quality development and the development of the insurance industry. We also identified the shortcomings in the current research and explained how our study addresses these gaps.

(8) Discussion and Conclusion:

The discussion section should delve deeper into the implications of the research findings and explore their implications for policy-making and practice in the insurance industry. The conclusion section should more clearly summarize the main findings of the study, highlight its limitations, and suggest directions for future research.

Thank you to the reviewers for their comments. In the conclusion section, we have elaborated on the research directions, identified research deficiencies, and outlined plans for further research. This addition aims to clarify our research positioning and future research agenda.(see lines 928 to 932 of the revised manuscript)

(9) Relevance of Supplementary Materials:

The supplementary materials mentioned in the paper should ensure they are closely related to the content of the main text and easy for readers to understand, avoiding unrelated or redundant information.

Thank you to the reviewer for your suggestions. In this revision, we have thoroughly reviewed the supplementary materials to enhance the readability of our research paper.

(10) Specific Policy Recommendations:

The paper should provide more specific policy recommendations to help decision-makers and practitioners understand how to promote the high-quality development of the insurance industry.

Thank you to the reviewer for your suggestions. We have enhanced the policy recommendations by including specific examples and suggestions from the perspective of insurance companies, thereby making our recommendations more concrete and actionable(see lines 895 to 898; lines 911 to 914).

**References**

[1] Yang, Y., & Zhang, P. (2021). Logic, Measurement, and Governance of High-Quality Development of China's Economy. Economic Research, 56(1), 26–42. from: <https://kns.cnki.net/kcms/detail/11.1081.F.20210406.1636.028.html>(in Chinese).
[2] Nie, C., & Jian, X. (2020). Measurement and Comparative Analysis of China's Regional High-Quality Development. Quantitative Economics & Technical Economics Research, 37(2), 26-47. doi: <https://doi.org/10.13653/j.cnki.jqte.2020.02.002>(in Chinese).
[3] Liu, Z., $ Re, Z. (2021). Regional differences and dynamic evolution of distribution of high-quality agricultural development in China. Quantitative Economics & Technical Economics Research, 38(6), 28-44. doi: <https://doi.org/10.13653/j.cnki.jqte.2021.06.002>(in Chinese).
[4] Li, H., & Qi, Y. (2022). Inclusive Insurance, Private Enterprises, and Common Prosperity: Analysis of Intermediary Effects and Adjustment Effects. Insurance Research, (8), 17–32. doi: <https://doi.org/10.13497/j.cnki.is.2022.08.002>(in Chinese).
[5] Zhang, L., Li, H., & Liang, Y. (2023). High-Quality Development of Rural Finance: Measurement and Spatiotemporal Evolution. China Rural Economy, 2023(1), 115-139. doi: <https://doi.org/10.20077/j.cnki.11-1262/f.2023.01.007>(in Chinese).
[6] Gao, Y., Xu, Y., Ji, C., et al. (2020). Measurement and Spatiotemporal Distribution Characteristics of High-Quality Development Level of China's Financial Industry. Quantitative Economics & Technical Economics Research, 37(10), 63–82. doi: <https://doi.org/10.13653/j.cnki.jqte.2020.10.004>(in Chinese).
[7] Xiong, J., & Tang, W. (2021). Impact of Homogenization of Insurance Business Structure on Systemic Risk of Property Insurance Industry. Insurance Research, (4), 43-61. doi: https://doi.org/10.13497/j.cnki.is.2021.04.003(in Chinese).
[8] Xiong, J., & Su, F. (2019). Measurement and Analysis of Guarantee Attributes of Insurance Companies. Insurance Research, (09), 44-59. doi: <https://doi.org/10.13497/j.cnki.is.2019.09.004>(in Chinese).
[9] Jiang, T., & Fan, J. (2017). Research on the Impact of Insurance Market Structure on Economic Growth—Based on Spatial Econometric Test of Provincial Panel Data. Technoeconomic Management Research, (03), 90-94. from: <https://kns.cnki.net/kcms2/article/abstract?v=wQLHse-RxfeRdC24H35Az0Xn8qRNdwL_HuzB27FJorsHthLbXUGKczOt8_8CrgTyzTlz_wqWXlw9efEhpubp5qxonlnwl83XYMaxVA9wG-p1BPE9fXYKwfY4l4EloBesfuA7NZEbzcepuV1werQb6g==&uniplatform=NZKPT&language=CHS>(in Chinese).
