# Supplementary material for: Spatio-temporal evolution and distribution characteristics of the high-quality development of China’s insurance industry
Source: PLoS One. 2025 Mar 4;20(3):e0311568. doi: 10.1371/journal.pone.0311568 (PMC11878946; doi:10.1371/journal.pone.0311568)
Supplement: Attachment — Submitted filename: revision.docx [file pone.0311568.s002.docx]

This study evaluates China's insurance sector development based on inclusiveness, sustainability, and structural coordination. It highlights significant regional disparities and imbalance, with the eastern region showing higher development but internal inequities, while the central and western regions demonstrate gradual convergence but subdued overall benchmarks. We commend the authors for their comprehensive study on evaluating China's insurance sector development. However, several key points need addressing:
1.In the last paragraph of the introduction, correct "Daugm's" to "Dagum's" Gini coefficient.
2.Introduce a new section titled "Materials and Methods" (Part 3), emphasizing the construction of the indicator system, along with data sources and calculation methodologies.
3.Merge the second half of Part 3 and Part 4 into the RESULTS section for better organization, focusing on the analysis and spatial distribution of high-quality development.
4.Clarify the methodology for dividing provinces into four levels based on quartile division and ensure consistency in table references (e.g., middle of 2013 and 2018 as 2015).
5.Consider extending the analysis in Section 4.4.2 with local Moran's index for a more comprehensive examination.
6.Incorporate a discussion section to provide insights and interpretations of the results.
7.Verify the format of figures depicting China's map and correct any discrepancies. Additionally, ensure accuracy in references and include international paper references.
Overall, a major revision is recommended to address these points thoroughly and enhance the clarity and coherence of the paper.

8.Table 7: Calculation Results of Dagum Gini coefficient. One of the year data in the Dagum Gini coefficient composition is incomplete. Please confirm

9.other details

(1).Introduction:
Further refine the marginal contribution of the article. There have been some studies on the measurement of the level of high-quality development of the insurance industry in the literature. How does this article differentiate from existing literature? The article also mentions " Currently, there is a lack of research on the construction of a comprehensive evaluation system for high-quality development in the insurance industry." Where does this point reflect? It is recommended that the author further elaborate and explain.
(2).Literature Review:
The content of the literature review is not focused enough, especially in the first part. It is not necessary to discuss the economic foundation of high-quality development from scratch, but rather to focus more on the review and evaluation of research on the high-quality development of the insurance industry in recent years. This will highlight the position of this article in existing literature and its innovations.
(3).Indicator Construction:
The article defines the high-quality development of the insurance industry based on the dimensions of "development" and "quality" as " the industry's development over a certain period, leading to changes in the satisfaction level of consumers' needs and values for insurance products and services." The focus is on consumers, but the indicator system does not reflect the concept of "consumer satisfaction."
(4).The article should clearly explain the data sources for indicator construction.
