# Supplementary material for: Spatio-temporal evolution and distribution characteristics of the high-quality development of China’s insurance industry
Source: PLoS One. 2025 Mar 4;20(3):e0311568. doi: 10.1371/journal.pone.0311568 (PMC11878946; doi:10.1371/journal.pone.0311568)
Supplement: Attachment — Submitted filename: Response to Reviewers.docx [file pone.0311568.s003.docx]

Dear Editor,

We sincerely thank you and all reviewers for the critical/valuable feedback. We feel lucky that our manuscript went to these reviewers as the valuable comments from them not only helped us with the improvement of our manuscript but suggested some neat ideas for future studies. Please do forward our heartfelt thanks to these experts.

Based on the comments we received, careful modifications have been made to the original manuscript. All changes were marked in yellow text and “Track Changes”. We hope the new manuscript will meet your standard. Below you will find out point-by-point responses to the comments/questions.

We look forward to hearing from you regarding our submission. We would be glad to respond to any further questions and comments that you may have.

Heng Zhu

(On behalf of all authors)

**Response to comments on “Spatio-temporal Evolution and Distribution Characteristics of the High-Quality Development of China's Insurance Industry (PONE-D-24-01860)”**

**Response to Editor Comments**

1.Please ensure that your manuscript meets PLOS ONE's style requirements, including those for file naming.

Thank you for your suggestion. We have checked the format of the manuscript and the file name.

2.Thank you for uploading your study's underlying data set. Unfortunately, the repository you have noted in your Data Availability statement does not qualify as an acceptable data repository according to PLOS's standards.

Thank you for your suggestion. We have organized all the data and code and uploaded them to the submission system.

3. We note that Figures 8, 9, 10 and 11 in your submission contain map images which may be copyrighted. All PLOS content is published under the Creative Commons Attribution License (CC BY 4.0), which means that the manuscript, images, and Supporting Information files will be freely available online, and any third party is permitted to access, download, copy, distribute, and use these materials in any way, even commercially, with proper attribution. For these reasons, we cannot publish previously copyrighted maps or satellite images created using proprietary data, such as Google software (Google Maps, Street View, and Earth).

Thank you for your suggestion. Due to the graphic format and copyright issues of Chinese maps, we have decided to delete map and submit only the "Basic Parameters of Standard Deviation Ellipse" table（Table 2 in the manuscript）. This table quantifies the results of the standard deviation ellipse method. While it is not as visually intuitive as directly displaying the map, the same conclusions can be drawn based on the specific data changes presented. If readers require the map results, we can provide them upon request.

**Response to Reviewer 1 Comments**

1.In the last paragraph of the introduction, correct "Daugm's" to "Dagum's" Gini coefficient.

Thanks for your careful checks. We acknowledge the oversight in our initial submission, where "Dagum's" was incorrectly written. We have corrected this error in the last paragraph of the introduction.

2.Introduce a new section titled "Materials and Methods" (Part 3), emphasizing the construction of the indicator system, along with data sources and calculation methodologies.

Thank you for your suggestion. In response to your request, we have added the "Materials and Methods" section to Part 3. This section details the calculation process for weighting each indicator and explains the data sources. Due to issues such as missing data in certain years and inconsistent statistical standards, some data required separate processing and calculations. Consequently, we have included detailed explanations and methods addressing the reasons for and processes used in handling such data. Meanwhile, we have attached the calculation code for comprehensive indicators.

3.Merge the second half of Part 3 and Part 4 into the RESULTS section for better organization, focusing on the analysis and spatial distribution of high-quality development.

Thank you for your suggestion. We have restructured Part 3, designating its second section as 4.1 to present the descriptive statistical results. Additionally, we have included an explanation of the method used to classify the 31 provinces into the East, Central, and West regions.（This classification is supported by the province division code, detailed in the "Dividing into regions" section of the Stata code. ）

4.Clarify the methodology for dividing provinces into four levels based on quartile division and ensure consistency in table references (e.g., middle of 2013 and 2018 as 2015).

Thank you for your suggestion. Our selection of quantiles was based on the overall data from 2010 to 2020, with quartiles selected for grouping (detailed quartiles have been added to line 493 of the article). We did not separately select quartiles from a representative year (such as 2014, 2015, or 2016) for grouping. The reasons are as follows:

Firstly, the interquartile range of the overall year is larger than that of a single year, as shown in Table 1, which can display the probability of changes in the high-quality development index of the insurance industry in each province on a larger scale. For example, Tables 2 and 3 show the traditional Markov matrix and spatial Markov matrix that divide the interquartile range based on 2015 and the overall year, respectively. The traditional Markov matrix includes regions that have developed from level I to level III. For the overall year, it has developed from below 0.3871 to 0.4363 to 0.4829, while for 2015, it has developed from 0.4140 to 0.4691. This indicates that the development level displayed is greater based on the overall year.

Secondly, the analysis in the previous part of the article indicates that the regional characteristics of high-quality development of China's insurance industry are characterized by significant differences between the two poles. Provinces with high development levels and those with low development levels coexist, making it more suitable to group them based on larger interquartile points.

Thirdly, there are significant differences between provinces with higher levels of development, especially in the eastern region. The non-parametric kernel density map shows a lack of "peaks", indicating that the development level is relatively scattered. However, even provinces with lower levels of development in the eastern region are better than those with higher levels of development in the central and western regions. For details, please refer to the Dagum Gini coefficient, where the contribution of super variable density is decreasing year by year. Therefore, if the number of grouping boundaries between the third and fourth groups selected is too low, it will not be possible to present the dynamic changes of regions with higher levels of development. For example, in 2016, the 75th percentile was 0.4951, which is higher than the overall 75th percentile of 0.4829. However, in 2016, both the 25th and 50th percentiles were too high, so the dynamic changes in areas with low levels of high-quality development in the insurance industry cannot be presented.

Based on the above considerations, we ultimately chose to group based on the quartiles of the overall year.

**Table 1: Overall Year and Representative Year Quantiles of the Insurance Industry High Quality Development Index**

|  | overall year | **2014** | **2015** | **2016** |
| --- | --- | --- | --- | --- |
| **25%quantile** | **0.3871** | **0.3649** | **0.4140** | **0.4346** |
| **50%quantile** | **0.4363** | **0.3965** | **0.4396** | **0.4616** |
| **75%quantile** | **0.4829** | **0.4350** | **0.4691** | **0.4951** |

**Table 2 Traditional Markov Matrix and Spatial Markov Matrix Dividing Quartile Points Based on 2015**

| **Spatial lag type** | **t/t+1** | **Ⅰ** | **Ⅱ** | **Ⅲ** | **Ⅳ** | **Frequency** |
| --- | --- | --- | --- | --- | --- | --- |
| **No-lag** | **Ⅰ** | **0.71875** | **0.148438** | **0.09375** | **0.039063** | **128** |
|  | **Ⅱ** | **0.254902** | **0.254902** | **0.313725** | **0.176471** | **51** |
|  | **Ⅲ** | **0.075472** | **0.207547** | **0.301887** | **0.415094** | **53** |
|  | **Ⅳ** | **0** | **0.012821** | **0.089744** | **0.897436** | **78** |
| **Ⅰ** | **Ⅰ** | **0.808511** | **0.117021** | **0.031915** | **0.042553** | **94** |
|  | **Ⅱ** | **0.368421** | **0.263158** | **0.263158** | **0.105263** | **19** |
|  | **Ⅲ** | **0.375** | **0.25** | **0.25** | **0.125** | **8** |
|  | **Ⅳ** | **0** | **0.111111** | **0** | **0.888889** | **9** |
| **Ⅱ** | **Ⅰ** | **0.529412** | **0.235294** | **0.235294** | **0** | **17** |
|  | **Ⅱ** | **0.363636** | **0.090909** | **0.181818** | **0.363636** | **11** |
|  | **Ⅲ** | **0** | **0.333333** | **0.5** | **0.166667** | **6** |
|  | **Ⅳ** | **0** | **0** | **0.166667** | **0.833333** | **6** |
| **Ⅲ** | **Ⅰ** | **0.466667** | **0.133333** | **0.333333** | **0.066667** | **15** |
|  | **Ⅱ** | **0.083333** | **0.333333** | **0.583333** | **0** | **12** |
|  | **Ⅲ** | **0** | **0.2** | **0.36** | **0.44** | **25** |
|  | **Ⅳ** | **0** | **0** | **0.25** | **0.75** | **16** |
| **Ⅳ** | **Ⅰ** | **0** | **1** | **0** | **0** | **2** |
|  | **Ⅱ** | **0.111111** | **0.333333** | **0.222222** | **0.333333** | **9** |
|  | **Ⅲ** | **0.071429** | **0.142857** | **0.142857** | **0.642857** | **14** |
|  | **Ⅳ** | **0** | **0** | **0.042553** | **0.957447** | **47** |

**Table 3 Traditional Markov Matrix and Spatial Markov Matrix for Dividing Quartile Points Based on overall year**

| **Spatial lag type** | **t/t+1** | **Ⅰ** | **Ⅱ** | **Ⅲ** | **Ⅳ** | **Frequency** |
| --- | --- | --- | --- | --- | --- | --- |
| **No-lag** | **Ⅰ** | **0.635294** | **0.329412** | **0.035294** | **0** | **85** |
|  | **Ⅱ** | **0.197674** | **0.348837** | **0.383721** | **0.069767** | **86** |
|  | **Ⅲ** | **0.035714** | **0.154762** | **0.452381** | **0.357143** | **84** |
|  | **Ⅳ** | **0** | **0** | **0.127273** | **0.872727** | **55** |
| **Ⅰ** | **Ⅰ** | **0.613636** | **0.386364** | **0** | **0** | **44** |
|  | **Ⅱ** | **0.259259** | **0.444444** | **0.259259** | **0.037037** | **27** |
|  | **Ⅲ** | **0** | **0.666667** | **0.333333** | **0** | **3** |
|  | **Ⅳ** | **0** | **0** | **0.25** | **0.75** | **4** |
| **Ⅱ** | **Ⅰ** | **0.741935** | **0.225806** | **0.032258** | **0** | **31** |
|  | **Ⅱ** | **0.322581** | **0.258065** | **0.354839** | **0.064516** | **31** |
|  | **Ⅲ** | **0.105263** | **0.157895** | **0.578947** | **0.157895** | **19** |
|  | **Ⅳ** | **0** | **0** | **0.25** | **0.75** | **4** |
| **Ⅲ** | **Ⅰ** | **0.444444** | **0.333333** | **0.222222** | **0** | **9** |
|  | **Ⅱ** | **0** | **0.380952** | **0.52381** | **0.095238** | **21** |
|  | **Ⅲ** | **0** | **0.152174** | **0.5** | **0.347826** | **46** |
|  | **Ⅳ** | **0** | **0** | **0.142857** | **0.857143** | **21** |
| **Ⅳ** | **Ⅰ** | **0** | **1** | **0** | **0** | **1** |
|  | **Ⅱ** | **0** | **0.285714** | **0.571429** | **0.142857** | **7** |
|  | **Ⅲ** | **0.0625** | **0.0625** | **0.1875** | **0.6875** | **16** |
|  | **Ⅳ** | **0** | **0** | **0.076923** | **0.923077** | **26** |

5.Consider extending the analysis in Section 4.4.2 with local Moran's index for a more comprehensive examination.

Thank you for your suggestion. We have conducted the local Moran's I test as per your suggestions and included the Moran scatter plots in Figures 8, 9, and 10 of the Figure file. During this revision process, we also re-conducted the global Moran's I test and found that the "internal-external structural coordination" dimension exhibits significant spatial autocorrelation, while the results for the "inclusiveness" and "sustainability" dimensions remain unchanged. These results have been incorporated into the revised manuscript.

Subsequently, we conducted a local Moran index test using data from the year 2020. The Moran scatter plots of the three dimensions showed no significant difference from the global Moran index results. The "inclusiveness" and "internal external structural coordination" dimension indices of most provinces in China were in the first and third quadrants, indicating a significant positive correlation.

6.Incorporate a discussion section to provide insights and interpretations of the results.

Thank you for your suggestion. Based on the analysis in the Results section, we systematically examined the temporal distribution, spatial distribution, dynamic transitions, and regional correlations of high-quality development in China's insurance industry. Next, we employ the Dagum Gini coefficient to further discuss and analyze the changes in spatial disparities and the main factors contributing to these differences in the high-quality development of China's insurance industry. Our findings reveal that the primary factor driving these disparities is regional differences, with inter-regional disparities being more significant than intra-provincial disparities, leading to a gradually evident gradient effect.

7.Verify the format of figures depicting China's map and correct any discrepancies. Additionally, ensure accuracy in references and include international paper references.

Thank you for your suggestion. Due to the graphic format and copyright issues of Chinese maps, we have decided to delete map and submit only the "Basic Parameters of Standard Deviation Ellipse" table（Table 2 in the manuscript）. This table quantifies the results of the standard deviation ellipse method. While it is not as visually intuitive as directly displaying the map, the same conclusions can be drawn based on the specific data changes presented. If readers require the map results, we can provide them upon request.

Additionally, we have revised the format of the literature review and added international paper. We have also added the DOIs of the references and the websites of the literature sources at the end of the references to ensure the accuracy and completeness of the literature review format.

8.Table 7: Calculation Results of Dagum Gini coefficient. One of the year data in the Dagum Gini coefficient composition is incomplete. Please confirm

Thank you for your suggestion.. We have carefully reviewed the results of the Dagum Gini coefficient decomposition and have added the missing 2015 section. Additionally, we have attached the Matlab code used for the Dagum Gini coefficient decomposition.

To ensure that the input data matches the spatial weight matrix, we used the "High Quality" data. This means that after calculating the comprehensive index results for each province, only the comprehensive index was retained. We then converted the long panel data into "wide panel data" and arranged it according to the order of the spatial weight matrix regions.

**Response to Reviewer 2 Comments**

(1).Introduction:

Further refine the marginal contribution of the article. There have been some studies on the measurement of the level of high-quality development of the insurance industry in the literature. How does this article differentiate from existing literature? The article also mentions " Currently, there is a lack of research on the construction of a comprehensive evaluation system for high-quality development in the insurance industry." Where does this point reflect? It is recommended that the author further elaborate and explain.

Thank you for your suggestion. In response, we have added detailed supplements to the last paragraph of the Introduction section regarding the marginal contributions of this paper. We have integrated the existing literature on measuring the high-quality development level of the insurance industry and further elaborated on the marginal contributions of this paper from various perspectives, including the starting point of measuring the high-quality development level of the insurance industry, theoretical interpretation of the indicators, and the considerations involved.

At present, research on the high-quality development of China's insurance industry is relatively limited, primarily characterized by the following aspects:

Lack of Industry-Level Studies: There is a scarcity of research at the industry level concerning the high-quality development of China's insurance industry. Existing studies predominantly focus on specific sectors within the insurance industry, such as agricultural insurance, health insurance, and life insurance. These studies often investigate the high-quality development of individual insurance products, leaving a gap in research that comprehensively examines the high-quality development of the entire insurance industry. For instance, Yuan Chunqing (2023), Yu Tianyi et al. (2024), Wang Wan (2023), Jia Hongbo et al. (2023), and Xu Xian et al. (2023) have conducted research in various dimensions of insurance, but there is limited examination from the perspective of the overall insurance industry.

Theoretical Deficiency: There is a lack of theoretical research concerning the high-quality development of the insurance industry. Existing studies tend to provide descriptive explanations without adequate grounding in relevant economic theories. There is a notable absence of theoretical frameworks elucidating the relationship between the high-quality development of the insurance industry and its historical development trajectory. Thus, this study seeks to address this gap by exploring theoretical frameworks derived from "quality" and "development" theories to underpin discussions on high-quality development within the insurance industry.

Insufficient Quantitative Research: Quantitative research on the high-quality development of the insurance industry is limited. While some studies, such as those by Hou Xuhua and Tang Yuhui (2022) and Jia Hongbo et al. (2023), have conducted quantitative explorations within specific segments of the insurance industry, there is a dearth of comprehensive quantitative analyses covering the entire insurance sector. This paper aims to contribute to this area of research by conducting quantitative investigations encompassing the entirety of the insurance industry.

In summary, there is a need for more comprehensive and theoretically grounded research at the industry level that utilizes quantitative methodologies to explore the high-quality development of China's insurance industry. This study endeavors to bridge these gaps by providing a holistic examination of high-quality development within the insurance sector.

(2).Literature Review:

The content of the literature review is not focused enough, especially in the first part. It is not necessary to discuss the economic foundation of high-quality development from scratch, but rather to focus more on the review and evaluation of research on the high-quality development of the insurance industry in recent years. This will highlight the position of this article in existing literature and its innovations.

Thank you for your suggestion. We have supplemented the literature review on the high-quality development of the insurance industry in the penultimate paragraph of the Introduction. A comprehensive review of the literature reveals that, on the one hand, the related studies on the high-quality development of the insurance industry predominantly focus on single product dimensions, rarely discussing the high-quality development level of the entire insurance industry as a starting point. On the other hand, when discussing the high-quality development level from the perspective of the insurance industry, existing research often exhibits limitations such as a narrow range of dimensions considered and a relatively weak theoretical foundation. Our study addresses these gaps and makes marginal contributions in these areas.

(3).Indicator Construction:

The article defines the high-quality development of the insurance industry based on the dimensions of "development" and "quality" as " the industry's development over a certain period, leading to changes in the satisfaction level of consumers' needs and values for insurance products and services." The focus is on consumers, but the indicator system does not reflect the concept of "consumer satisfaction."

Thank you for your suggestion. Indeed, including an indicator of "consumer satisfaction" would provide a more comprehensive understanding of the level of high-quality development within China's insurance industry. Consumer satisfaction is a critical aspect of assessing the quality of services provided by insurance companies and their overall performance in meeting customer needs and expectations.

It's understandable that this indicator may not be readily available in official insurance industry data in China. However, conducting field research to collect data on consumer satisfaction would be a valuable endeavor for future studies. By directly gathering feedback from insurance consumers through surveys, interviews, or other research methods, insights into consumer perceptions, preferences, and satisfaction levels can be obtained. This data could then be integrated into the analysis to provide a more nuanced assessment of the high-quality development of China's insurance industry.

Incorporating consumer satisfaction as an indicator in future research would not only enhance the comprehensiveness of the analysis but also contribute to a deeper understanding of the relationship between the insurance industry's performance and consumer demand. Thank you for considering this important aspect for future study. In the future, we will collect this data through field research.

(4).The article should clearly explain the data sources for indicator construction.

Thank you for your suggestion. We have explained the data sources for selecting indicators in Part 3 as per your request. Additionally, due to missing data, inconsistent statistical methods, and the need for special handling, we have also provided relevant explanations for the processing of this type of data in Part 3.

**References**

[1] Yuan, Q. (2023). High-Quality Development of Agricultural Insurance Must Adhere to Six Orientations. Insurance Research, (05), 3-8. doi: <https://doi.org/10.13497/j.cnki.is.2023.05.001>(in Chinese).
[2] Yv, T., Lv, K., & Zhang, C. (2024). Promote the High-Quality Development of Fishery Insurance. Macroeconomic Management, (05), 75-81+92. doi: <https://doi.org/10.19709/j.cnki.11-3199/f.2024.05.012>(in Chinese).
[3] Wang, W. (2023). Commercial Health Insurance in China: Demand Satisfaction, Market Rules and High-Quality Development. Academic Research, (10), 94-100+177. from: <https://kns.cnki.net/kcms2/article/abstract?v=K_cp52o2S79Ye9kwL5_h6Vw6aAik9myf8jc36sRjAe7j8hQcwJUodDJ7v9ZdM1GobaIJVVdGBZCSlV7qpM8-dYbBtGURD1I9AakmoXtsu9yyUAjLKMxsPcs5NaVy8WXKikxixiB41zjLG89b0EnSbw==&uniplatform=NZKPT&language=CHS>(in Chinese).
[4] Jia, H., Cai, Y., & Tang z. (2023). Regional Differences and Formation Mechanisms of High-Quality Development of Basic Medical Insurance in China—An Empirical Study Based on Provincial Panel Data From 2011 to 2020. Social Security Research, (06), 3–19. from: <https://kns.cnki.net/kcms2/article/abstract?v=K_cp52o2S7-8is46TwOdi5DvR_O8rZGqrcmc29W90ITxPGM8TBvl57uezhj2uyxhlyVXsWqSVP16vlKMXRJ2NQJaHvoPmfgSzEC7mh_Q6MUAkvBGwf-hp1KYTWCNFq-zXeJb45h_I5t0Sktmwvvb9A==&uniplatform=NZKPT&language=CHS>(in Chinese).
[5] Xu, X., Luo, J., & Wei, J. (2023). High-Quality Development of The Life Insurance Indus-try and Insurance Agent Management—An Analysis Based on The Perspective of The “B-asic Law” of The Life Insurance Industry. Insurance Research, (02), 34-44+89. doi: <https://doi.org/10.13497/j.cnki.is.2023.02.003>(in Chinese).
[6] Hou, X., & Tang, Y. (2022). Evaluation Index Design and Measurement Research on the High-Quality Development of Internet Insurance Companies. Insurance Research, (09), 78–90. doi: <https://doi.org/10.13497/j.cnki.is.2022.09.007>(in Chinese).
[7] Huang, F., Zhuo, Z., & Wu, C. (2015). Research on Insurance Customer Satisfaction - Analysis of Influencing Factors and Customer Segmentation Based on the Life Insurance Market. Insurance Research, (09), 9–22. doi: <https://doi.org/10.13497/j.cnki.is.2015.09.002>(in Chinese).
[8] Zhang, L., & He, B. (2023). Regional Differences and Spatial Convergence of High-Quality Development of Agricultural Insurance—Theoretical Interpretation of the High-Quality Development Perspective in the Report of the 20th National Congress of the Communist Party of China. Financial Economics Research, 38(01), 113-128. from: <https://kns.cnki.net/kcms/detail/44.1696.F.20230116.1534.005.html>(in Chinese).
[9] Chen, W., & Li, C. (2023). Measurement, Regional Differences and Structural Decomposition of High-Quality Development Level of Social Security in My Country. Chinese Administration, 39(12), 83-95. doi: <https://doi.org/10.19735/j.issn.1006-0863.2023.12.10>(in Chinese).
[10] Huraini, Thaer.Towards the development of agricultural insurance in Palestine in considering the challenges of water and land[J].AGUA Y TERRITORIO,2024,(24). doi: <https://doi.org/10.17561/at.24.7818>.
[11] Akinlo, Taiwo.Information technology and insurance development in Sub-Saharan Africa.[J].Information Development,2023,Vol.39(1): 169-183. doi: <https://doi.org/10.1177/02666669211028960>.
[12] Zhichkin, Kirill A.;Nosov, Vladimir V.;Zhichkina, Lyudmila N..Agricultural Insurance, Risk Management and Sustainable Development[J].Agriculture (Switzerland),2023,Vol.13(7): 1-3. doi: <https://doi.org/10.3390/agriculture13071317>.
[13] Tatiana DZIUBETCAIA;Natalia COSTOVA.POSITION AND DEVELOPMENT OF THE MOLDOVAN INSURANCE MARKET[J].Economica,2022,Vol.3(121): 91-103. doi: <https://doi.org/10.53486/econ.2022.121.091>.
[14] Yonglian Chang, Yingjun Huang, Manman Li, Zhengmin Duan.The effect of haze pollution on insurance development: Evidence from 268 prefecture-level cities in China[J].PloS one,2022,Vol.17(4): e0267830. doi: <https://doi.org/10.1371/journal.pone.0267830>.
[15] Yan Zhang, Wenxi Tang, Xiang Zhang, Yaoguang Zhang, Liang Zhang.National Health Insurance Development in China from 2004 to 2011: Coverage versus Benefits[J].PloS one,2015,Vol.10(5): e0124995. doi: <https://doi.org/10.1371/journal.pone.0124995>.
