# Supplementary material for: Spatio-temporal evolution and distribution characteristics of the high-quality development of China’s insurance industry
Source: PLoS One. 2025 Mar 4;20(3):e0311568. doi: 10.1371/journal.pone.0311568 (PMC11878946; doi:10.1371/journal.pone.0311568)
Supplement: Attachment — Submitted filename: Dear Editor in Chief and Professor.docx [file pone.0311568.s004.docx]

**Dear Editor in Chief and Professor**

For this article, I suggest that the author correct it as follows

(a)The derivation of equations (1) to (9) does not seem to be connected to the content
of Table 3 in the article.

(b)In addition, the empirical results in Table 3 do not include the variable self-
attribution test, the single root test, the co-integration test, and other pre-statistical tests, causality tests, etc.

(c)In addition, please confirm whether the 2011 and 2012 years of Inclusiveness in
Table 3 should be 2015 and 2020

(d) There are too few references and it is not detailed whether the formula was created by the author himself or a reference, and the author is asked to explain why formulas (1) to (9) are used, and whether the model has any shortcomings, rather than other more suitable models to analyze

Sincerely Yours

Academic Editor

Professor Dr. Yu kun Wang

Date; 2024/8/28
