# Supplementary material for: Spatio-temporal evolution and distribution characteristics of the high-quality development of China’s insurance industry
Source: PLoS One. 2025 Mar 4;20(3):e0311568. doi: 10.1371/journal.pone.0311568 (PMC11878946; doi:10.1371/journal.pone.0311568)
Supplement: Attachment — Submitted filename: decision.docx [file pone.0311568.s006.docx]

1.I would like to ask: The numerator of formula 20 has two identical i=1 to N, which seems to be wrong Please explain
