# Supplementary material for: Spatio-temporal evolution and distribution characteristics of the high-quality development of China’s insurance industry
Source: PLoS One. 2025 Mar 4;20(3):e0311568. doi: 10.1371/journal.pone.0311568 (PMC11878946; doi:10.1371/journal.pone.0311568)
Supplement: Attachment — Submitted filename: Response_to_Reviewers_auresp_3.docx [file pone.0311568.s007.docx]

Dear Editor,

We sincerely thank you and all reviewers for the critical/valuable feedback. We feel lucky that our manuscript went to these reviewers as the valuable comments from them not only helped us with the improvement of our manuscript but suggested some neat ideas for future studies. Please do forward our heartfelt thanks to these experts.

Based on the comments we received, careful modifications have been made to the original manuscript. All changes were marked in yellow text and “Track Changes”. We hope the new manuscript will meet your standard. Below you will find out point-by-point responses to the comments/questions.

We look forward to hearing from you regarding our submission. We would be glad to respond to any further questions and comments that you may have.

Heng Zhu

(On behalf of all authors)

**Response to Academic Editor Comments**

1.I would like to ask: The numerator of formula 20 has two identical i=1 to N, which seems to be wrong Please explain

Thank you very much for the editor's comments. Due to an error, we mistakenly included a summation symbol when writing the formula. The correct form of formula 20 should be as follows:

$$\begin{aligned} S^{2}=\frac{\sum_{i=1}^{n} \left( x_{i}-\bar{x} \right)^{2}}{n}\#\left( 20 \right) \end{aligned}$$

The calculation of $S^{2}$ is based on sample variance. We have made the necessary correction on line 740 (Formula 20) of the revised manuscript.

In addition, we sincerely apologize for this mistake. We have reviewed and checked the formulas throughout the text to ensure their accuracy. We apologize once again and greatly appreciate your understanding.

**Response to** **Journal Requirements:**

1. Please review your reference list to ensure that it is complete and correct. If you have cited papers that have been retracted, please include the rationale for doing so in the manuscript text, or remove these references and replace them with relevant current references. Any changes to the reference list should be mentioned in the rebuttal letter that accompanies your revised manuscript. If you need to cite a retracted article, indicate the article’s retracted status in the References list and also include a citation and full reference for the retraction notice.

Thank you very much for the editor's comments. Upon reviewing the citations in the full text, we found that some references were inadvertently omitted during the revision process. In this revision, we have supplemented the missing references and indicated their citation positions in the text (see lines 67 and 543 of the revised manuscript). We have also rechecked the reference format and made corresponding adjustments.

2. While revising your submission, please upload your figure files to the Preflight Analysis and Conversion Engine (PACE) digital diagnostic tool, [https://pacev2.apexcovantage.com/](https://c05y1x9s.r.us-east-2.awstrack.me/L0/https:%2F%2Fpacev2.apexcovantage.com%2F/1/010f0191e473d498-215a0baf-ccb5-4c5a-92e0-be46ead8d552-000000/z4fCoiq8t_eRCS1lgGFp8gI_pJk=176). PACE helps ensure that figures meet PLOS requirements. To use PACE, you must first register as a user. Registration is free. Then, login and navigate to the UPLOAD tab, where you will find detailed instructions on how to use the tool. If you encounter any issues or have any questions when using PACE, please email PLOS at [figures@plos.org](mailto:figures@plos.org). Please note that Supporting Information files do not need this step.

Thank you very much for the editor's comments. We have upload our figure files to the Preflight Analysis and Conversion Engine (PACE) digital diagnostic tool, and ensure that figures meet PLOS requirements. We will place the latest images in the figure. word file and upload to the system. The following figure shows the image processing record:

Figure 1 Image Processing Record
